# Comparative impact of proton versus photon irradiation on triple‐negative breast cancer: Role of VEGFC in tumour aggressiveness

**DOI:** 10.1002/ctm2.70330

**Published:** 2025-05-21

**Authors:** Saharnaz Sarlak, Delphine Marotte, Arthur Karaulic, Jessy Sirera, Alessandra Pierantoni, Meng‐Chen Tsai, Roxane Sylvestre, Clement Molina, Arthur Gouraud, Aurélien Bancaud, Paraskevi Kousteridou, Marie Vidal, Joël Hérault, Jérôme Doyen, Maeva Dufies, Florent Morfoisse, Barbara Garmy‐Susini, Frédéric Luciano, Gilles Pagès

**Affiliations:** ^1^ University Cote d'Azur (UCA), Institute for Research on Cancer and Aging of Nice (IRCAN) Centre Antoine Lacassagne Nice France; ^2^ I2MC Université de Toulouse Toulouse France; ^3^ CNRS LAAS Toulouse France; ^4^ Centre de Recherche en Cancérologie de Marseille (CRCM) Marseille France; ^5^ Institut Méditerranéen de Protonthérapie Centre Antoine Lacassagne Fédération Claude Lalanne Nice France

1

Dear Editor,

In this study, we demonstrated that proton (P) and photon (X) radiotherapies (RT) lead to different molecular changes in triple‐negative breast cancer (TNBC) cells. P‐irradiated tumours tended to make larger tumours, while X‐irradiated ones exhibited increased aggressiveness. Both types of radiation increased gene expression related to angiogenesis (blood vessel formation) and lymphangiogenesis (lymph vessel formation), which are associated with more aggressive cancer behaviour. We also found that targeting the lymphangiogenesis‐related gene, vascular endothelial growth factor C (VEGFC), alongside either type of RT, could improve the prognosis for TNBC patients.

Breast cancer (BC) is the most common type of cancer among women.^1^ Its aggressive forms, like TNBC, tend to be highly vascularized and often have an increased network of lymphatic vessels, which allows the cancer to metastasize more rapidly.^2^ Standard treatment involves with a combination of surgery, chemotherapy and RT to target both local and systemic diseases. Despite these treatments, recurrence remains a significant challenge in aggressive forms of BC.^3^

Proton therapy, a newer form of RT, offers more precise targeting than conventional X‐RT, potentially reducing side effects by narrowing the radiation field.^4^ Ongoing clinical trials are investigating whether P‐RT might offer advantages over conventional X‐RT, as recent research suggests promising advantages.^5^

Here, we investigated how irradiation impacts TNBC cell behaviour and their microenvironment, building on our prior study of P‐ and X‐RT effects on head and neck cancer.^6^ Specifically, we investigated whether irradiation might inadvertently promote tumour growth by altering cells to release growth factors or cytokines that support tumour survival and progression.

To examine these effects, we developed TNBC cell populations (MDAMB231 and BT549) that are resilient to repeated X‐ or P‐RT. The traits of aggressiveness, such as proliferation and migration were evaluated in these multi‐irradiated cells. While proliferation rates in irradiated cells were like controls (Figure 1A,B), migration abilities were enhanced (Figure 1C,D), suggesting that these cells could have a greater potential for metastasis. This increase in migration mirrors findings in X‐resistant medulloblastoma cells.^7^

**FIGURE 1:**
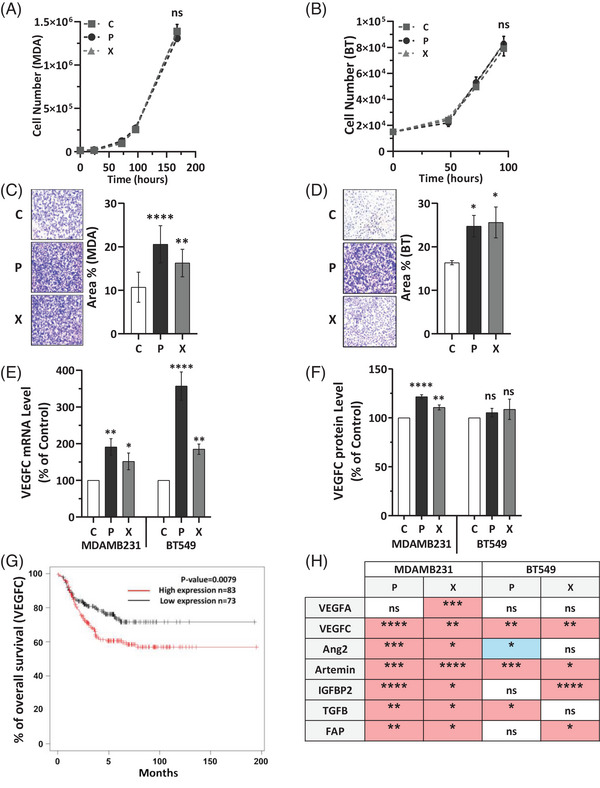
Multi‐irradiated triple‐negative breast cancer (TNBC) cells acquired pro‐metastatic properties and higher VEGFC production. (A, B) The proliferation rate (cell counts) of MDAMB231 and BT549 cells was evaluated following seven rounds of P and X irradiations (8 Gy), referred to as multi‐irradiated cells, compared to their respective controls (C, 0 Gy). (C, D) The migration ability of multi‐irradiated (P and X) BT549 and MDAMB231 was assessed compared to their corresponding controls. (E) Evaluation of VEGFC mRNA levels of multi‐irradiated MDAMB231 and BT549 cells with P or X irradiations, compared to their corresponding controls, using quantitative PCR. (F) Quantification of secreted VEGFC protein levels in the supernatant of P and X multi‐irradiated MDAMB231 and BT549 cells compared to their respective controls (C) using ELISA. (G) Kaplan–Meier analysis of overall survival (OS) of TNBC patients using the Kaplan–Meier software (https://kmplot.com/analysis/). OS was calculated from patient subgroups with mRNA levels of VEGFC that were less or greater than the best cut‐off value. (H) Quantitative gene expression analysis of P and X multi‐irradiated MDAMB231 and BT549 compared to the corresponding control (C). Red indicates upregulation of the gene compared to the respective control, while blue represents downregulation. The results are presented as the mean of at least three independent experiments ± SD. Statistical analysis was performed using one‐way ANOVA to compare differences between the control and irradiated groups (P or X). Statistical significance is denoted as follows: *p < .05, **p < .01, ***p < .001, ****p < .0001. NS, non‐significant.

Since metastasis in TNBC frequently occurs via lymphatic vessels,^8^ we investigated the impact of X‐ and P‐RT on the expression of VEGFC, a key regulator of lymphangiogenesis, in our TNBC cell lines which exhibit higher basal levels of VEGFC compared to cell lines of other BC subtypes (Figure S1). Both irradiation types significantly upregulated VEGFC mRNA expression (Figure 1E) and increased secretion of VEGFC protein (Figure 1F), (similar trend in BT549 cells). A comparable increase in VEGFC mRNA levels was also noted when comparing single versus multiple rounds of RT (Figure S2). Higher VEGFC expression has been associated with worse clinical outcomes in TNBC, as evidenced by patient survival data from existing databases (KM plotter software) (Figure 1G), suggesting that both baseline VEGFC levels and RT‐induced VEGFC upregulation could contribute to worse prognosis in TNBC. This elevated VEGFC expression indicates a potential risk of enhanced lymphangiogenesis, and thus higher recurrence and metastasis.^9^

Other genes linked to poor survival, such as artemin, angiopoietin 2, IGFBP2, FAP and TGFβ were also differentially expressed after irradiation in both cell lines, further suggesting that radiation may subtly alter gene expression in ways that could increase relapse risk (Figure 1H). Individual data for each factor are presented in Figure S3. These findings emphasize the need to consider the distinct impacts of different radiation types on the tumour cells and the surrounding microenvironment when devising TNBC treatment strategies.

Furthermore, the aggressiveness and the capacity of the multi‐irradiated cells to promote metastasis via lymphatic vessels was evaluated by permeability assay. Vessel‐on‐chip experiments using reconstituted lymphatic vessels surrounded by multi‐irradiated X‐irradiated MDAMB231 cells demonstrated increased vessel leakiness compared to vessels exposed to either control or multi‐irradiated P‐irradiated MDAMB231 cells (Figure 2A,B), suggesting enhanced metastatic potential. Considering the aggressive behaviour observed in vitro in TNBC cells adapted to repeated irradiations, we conducted experiments to assess the tumourigenic capacity of TNBC P‐ and X‐adapted cells in nude mice. Tumours from P‐adapted cells had higher incidence (100%) compared to 60% in X‐adapted, and 40% in controls (Figure 2C) and were larger than those from X‐adapted or control cells (Figure 2D,E). However, X‐irradiated tumours, though smaller, showed higher expression of lymphatic (Lyve1) and vascular markers (CD31), indicating a more aggressive molecular profile than P‐irradiated tumours (Figure 2F–I). Moreover, VEGFC protein expression was elevated in X‐irradiated tumour lysates compared to both P‐irradiated and control (C) tumours (Figure 2J). Transcriptomic analysis performed on human genes (tumours cells) and mouse genes (microenvironment) revealed that X tumours had more active pathways for angiogenesis, lymphangiogenesis and other genes associated with aggressiveness such as those involved in epithelial‐mesenchymal transition (Figure S4A,B). This suggests that X‐irradiated tumours may carry a higher risk of aggressive relapse.

**FIGURE 2:**
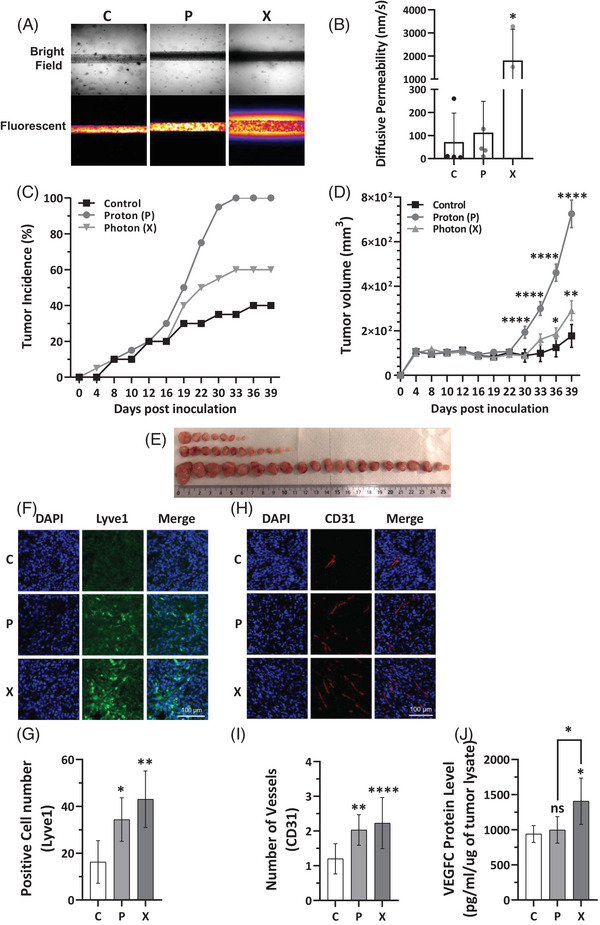
Triple‐negative breast cancer (TNBC) cells subjected to multiple rounds of X‐irradiation exhibit features associated with increased tumour aggressiveness. (A) 3D microvessels were reconstituted in collagen gel using MDAMB231 tumour cells (control, P‐irradiated, and X‐irradiated) within in‐house fabricated polydimethylsiloxane (PDMS)‐based chips. Imaging was performed using both brightfield and fluorescence microscopy (FITC/488), with visualization rendered via Imaji Studio Fire representation. (B) Diffusive permeability measurements of liquid in lymphatic vessels, constructed from LECs and surrounded by MDAMB231 cells (control, P‐irradiated, and X‐irradiated), were performed to assess vessel integrity. The evaluation of tumours generated following the xenografting of either non‐irradiated (Control), seven rounds of P‐irradiated (8 Gy), or X‐irradiated (8 Gy) MDAMB231 cells in immunodeficient mice. (C) Comparison of tumour incidence between the control, P and X groups under the same conditions. (D) Monitoring of tumour growth in the three groups, including control and irradiated groups, over a period of 39 days. (E) Representative image of the tumour xenografts showcasing the morphology and growth of the tumours in each experimental group. Immunofluorescence (IF) of lymphatic and vascular markers in experimental tumours generated with control (C), multi‐irradiated X (X) and P (P) MDAMB231 cells. (F, G) Representative images of LYVE1 (lymphatic endothelial cells, green)/Hoechst (nuclei, blue) staining, showing different patterns of lymphatic vessels development in P, X and C tumour groups. (H, I) Representative images CD31 (endothelial cells, red)/Hoechst (nuclei, blue) staining, showing anarchic blood vessels structures in P, X and C tumour groups. (J) VEGFC protein level in C, P and X tumour lysates. Differences between the control and irradiated groups (P or X) were analysed using one‐way ANOVA. Statistical significance was denoted as follows: *p < .05, **p < .01, ***p < .001, ****p < .0001. NS, non‐significant.

Proteomic analysis of the tumour tissues revealed distinct molecular profiles between the two irradiation types (Figure S5A). The unique protein signatures in P‐ and X‐irradiated tumours were confirmed using Principal Component Analysis (PCA), which showed clear separation between the two types based on their protein expression (Figure S5B). This result underscores that P‐ and X‐RT impact TNBC tumour biology in different ways.

To investigate the role of VEGFC in radiation response, we used CRIPRS/Cas9 to knockout the VEGFC gene in MDAMB231 and BT549 cell lines (Table S1, Figure S6). VEGFC‐deficient cells (VEGFC‐/‐) had significantly lower survival rates after X‐ or P‐RT compared to controls, with almost no viable VEGFC‐/‐ cells remaining (Figure 3A–D). However, VEGFC knockout did not affect the cells' response to chemotherapy, indicating VEGFC's specific influence on RT sensitivity (Figure S7A–C). Furthermore, VEGFC knockout did not affect cell proliferation in vitro (Figure 3E,F). To assess the role of VEGFC in modulating sensitivity to RT, MDAMB231 cells were exposed to high‐dose RT (8 Gy) with or without preincubation with recombinant VEGFC protein. While both X‐ and P‐RT significantly reduced cell numbers, preincubation with VEGFC conferred a protective effect (Figure S8). This finding is consistent with our previous observation that VEGFC‐/‐ cells exhibit heightened sensitivity to RT.

**FIGURE 3:**
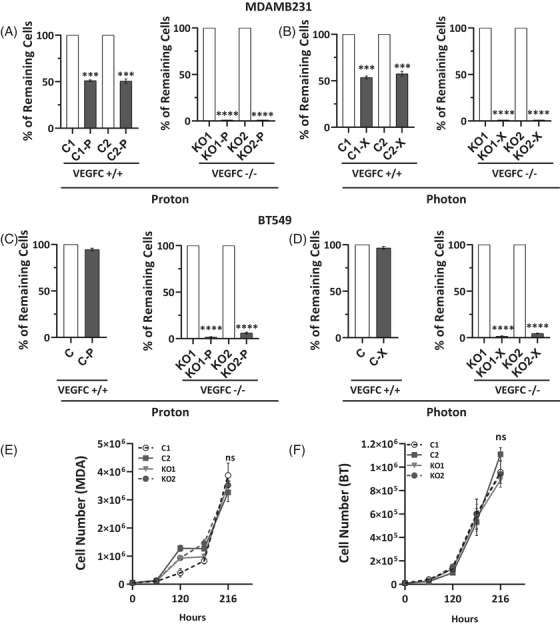
VEGFC knock‐out cells are more sensitive to irradiation. (A, B) Following 2 weeks of exposure to P or X irradiation (8 Gy), cell count was performed on CRISPR/Cas9‐edited MDAMB231 cells, with the empty vector serving as the control (C) and VEGFC knockout (KO) as the experimental condition. One clone from the control group and two clones were assessed for each condition. (C, D) Following two weeks of exposure to P or X irradiation (8 Gy), cell count was performed on CRISPR/Cas9‐edited BT549 cells, with the empty vector serving as the control (C) and VEGFC knockout (KO) as the experimental condition. One clone from the control group and two clones from the KO group were assessed for each condition. (E, F) Cell counts of CRISPR/Cas9‐edited MDAMB231 and BT549 cells, including control (C) and knockout (KO) variants, were monitored over a period of 216 h. Two clones were evaluated for each condition. The results are presented as the mean of at least three independent experiments ± SD. Statistical analysis was performed using one‐way ANOVA to compare differences between the control and irradiated groups (P or X). Statistical significance is denoted as follows: *p < .05, **p < .01, ***p < .001, ****p < .0001. NS, non‐significant.

In a related in vitro experiment, treatment with an anti‐VEGFC antibody reduced cell counts by up to 90% in both control and multi‐irradiated X‐ and P‐irradiated cells, suggesting that endogenous and RT‐induced VEGFC function as an autocrine factor promoting proliferation and/or survival (Figure 4A,B). Anti‐VEGFC antibody treatment inhibited the growth of experimental tumours in nude mice derived from control as well as multi‐irradiated X‐ and P‐irradiated MDAMB231 cells, with some tumours exhibiting near‐complete regression (Figure 4C–E). This suggests that anti‐VEGFC therapy, especially alongside RT, could be an effective strategy to limit TNBC progression.

**FIGURE 4:**
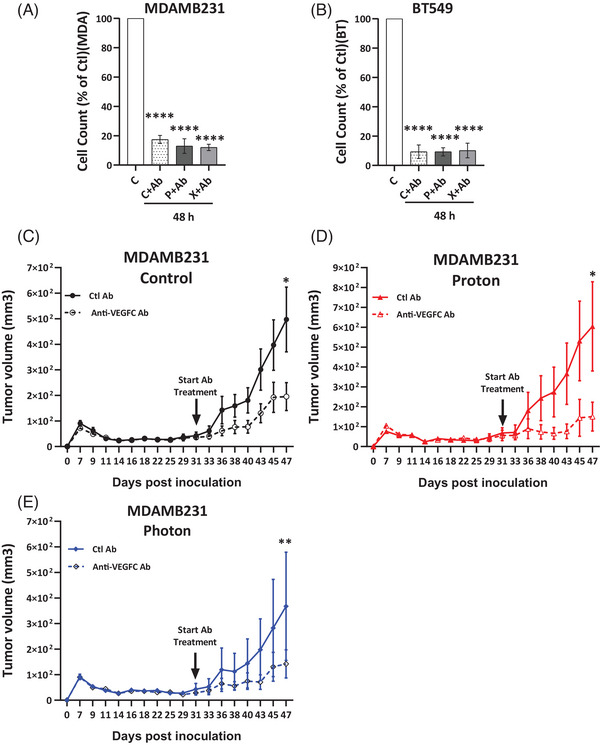
Anti‐VEGFC antibodies slow down the growth of experimental triple‐negative breast cancer (TNBC). (A, B) Effect of anti‐VEGFC antibody on MDAMB231 and BT549 multi‐irradiated for 48 h. (C–E) The evaluation of tumours generated following xenografting of MDAMB231 (C) Control, (D) P‐ and (E) X‐multi‐irradiated cells in immunodeficient mice over a period of 47 days. Starting from day 30th post‐injection of 7 × 10^6^ tumour cells subcutaneously, half of the mice in each group received anti‐VEGFC antibody (7.5 mg/kg twice a week), while the other half received an irrelevant antibody (Control), for a total duration of 17 days. Differences between the control and irradiated groups (P or X) treated with antibody were analysed using one‐way ANOVA (A, B). Differences between the control and treated groups were analysed using the Student's *t*‐test (C–E). Statistical significance was denoted as follows: *p < .05, **p < .01, ***p < .001, ****p < .0001. NS, non‐significant.

In conclusion, our study reveals that P and X radiotherapies produce different molecular effects in TNBC cells, with P‐irradiated tumours being larger and X‐irradiated tumours displaying more aggressive molecular characteristics. These differences suggest that X‐RT may lead to more aggressive relapses if resistant cells survive. Future research should focus on understanding these radiation‐specific effects and developing strategies to manage risks associated with X‐RT. Furthermore, our study emphasizes the potential of anti‐VEGFC therapies, especially if administered before irradiation, to counteract radiation‐induced molecular changes. Comparative clinical trials and further investigations into anti‐VEGFC treatment schedules are anticipated to advance TNBC treatment strategies.

## AUTHOR CONTRIBUTIONS

*Conception and design*: Gilles Pagès and Frédéric Luciano. *Development of methodology*: Saharnaz Sarlak. *Acquisition of data*: Saharnaz Sarlak, Delphine Marotte, Alessandra Pierantoni, Jessy Sirera, Meng‐Chen Tsai, Arthur Karaulic and Roxane Sylvestre. *Analysis and interpretation of data*: Saharnaz Sarlak, Florent Morfoisse, Barbara Garmy‐Susini, Frédéric Luciano, Gilles Pagès and Paraskevi Kousteridou. *Writing, review*: Saharnaz Sarlak, Florent Morfoisse, Meng‐Chen Tsai, Frédéric Luciano and Gilles Pagès. *Administrative, technical, or material support*: Marie Vidal, Joël Hérault and Gilles Pagès. *Study supervision*: Frédéric Luciano and Gilles Pagès.

## CONFLICT OF INTEREST STATEMENT

The authors declare no conflicts of interest.

### ETHICS STATEMENT

This study was carried out in strict accordance with the recommendations in the Guide for the Care and Use of Laboratory Animals. Our experiments were approved by the “Comite National Institutionnel d'Ethique pour l'Animal de Laboratoire (CIEPAL)” (reference: NCE/2023823).

## Supporting information



Supporting Information

Supporting Information

Supporting Information

## Data Availability

All data generated or analysed during this study are available from the corresponding author upon reasonable request.
