# Supplementary material for: Comparative impact of proton versus photon irradiation on triple‐negative breast cancer: Role of VEGFC in tumour aggressiveness
Source: Clin Transl Med. 2025 May 21;15(5):e70330. doi: 10.1002/ctm2.70330 (PMC12095174; doi:10.1002/ctm2.70330)
Supplement: Supplementary file 1 — Supporting Information [file CTM2-15-e70330-s002.docx]

**Materials and Methods**

***Cell Lines and Culturing Conditions***

Human triple negative breast cancer cell lines (MDAMB231, BT549) were purchased from the ATCC. These cells were cultured in DMEM (1X) + GlutaMAX 4.5 g/L D-Glucose (Cat. N. 10566016, Gibco®) supplemented with 10% Fetal Bovine Serum (FBS) (Cat. N. 10270106, Gibco®), 1% MEM Non-Essential Amino Acids Solution, (Cat. N. 11140050m, Gibco®) and 1% penicillin-streptomycin (Gibco®). All the cells maintained at 37 °C in humidified atmosphere at 5% CO2.

All cell lines were regularly tested for mycoplasma contamination (every 4 weeks) using Mycoplasma Detection Kit, and were found to be negative throughout the course of the experiments

VEGFCKO clones: The VEGFC gene was knocked-out in wild type MDAMB231 and BT549 cells by the CRISPR-Cas9 technique (1). Briefly, a human *VEGFC* target oligonucleotide (5′-GAGTCATGAGTTCATCTACAC-3′) was cloned into the pX330-U6-Chimeric_BB-CBh-hSpCas9 vector (Addgene plasmid # 42230). Two *VEGFC*^KO^ clones were obtained by PEI transfection (Tebu Bio, Le-Perray-en-Yvelines, FRANCE) of the resulting vector into MDAMB231 or BY549 cells and further selection on 5 µg/ml puromycin (InvivoGen, Toulouse, France), for 10–15 days. Control cells (Ctl) were obtained by transfection of WT-MDAMB231 or WT-BT549 cells by an empty pX330 vector and puromycin selection. The mutations leading to *VEGFC* invalidation were revealed, for each clone, by genomic DNA sequencing, using the following primers: Sense, 5′-TTGTGTTAGGGAACGGAGCAT-3′; Antisense, 5′-AGAACCAGGCTGGCAACTTC-3′ (Supp. Table S1). Effectiveness of *VEGFC* invalidation was confirmed by ELISA assay of VEGFC production.

***Cell irradiations***

Hundred fifty thousand cells were seeded in the 12 cm^2^ tissue culture flasks, 24 h prior to the irradiations. Proton irradiation was done at Centre Antoine Lacassagne, Nice (CAL) using 63 MeV Cyclotron MEDICYC and Photon irradiation was done using a Faxitron cabinet X-ray irradiator (160kV-6.3 mA; Edimex, Le Plessis-Grammoire, FRANCE) in 2, 4 and 8 Gy.

To generate multi-irradiated cells, MDAMB231 and BT549 cells were subjected to repeated irradiation rounds. Approximately 3–4 weeks after each exposure -once the cells had sufficiently recovered- they were reseeded following the initial protocol and irradiated again. This process was repeated seven times to produce the final population of multi-irradiated cells.

***Cell proliferation***

Cells were seeded in six-well plates in triplicates and were counted every day or every 48 h for 6 to 8 days, using a Coulter counter (Villepinte, FRANCE).

***Transmigration***

The transmigration assay based on chemotaxis was performed by inserting a transwell polyester membrane filter with 8μm pores polycarbonate membrane (Falcon® Cell Culture Inserts. Product number: 353097) in 24-well culture plates. Total of 150 000 serum starved MDAMB231, BT549 and TIME cells were resuspended in 300μL of the serum free corresponding media seeded in upper chamber of Transwell. To chemoattract the cells, the corresponding medium containing 10% FBS was placed in the lower chamber. After 24h of incubation, the migrated cells on the bottom side of the membrane were counted. Each assay was performed in triplicate wells. Cells were counted using the ImageJ software (NIH; Bethesda, MD, USA).

***Enzyme-linked immunosorbent assay (ELISA)***

The conditioned media of cells 48 h post seeding were collected, and the corresponding cells were counted for data normalization. VEGFC was quantified from supernatants with the R&D Systems Human VEGFC Duoset ELISA kit (Minneapolis, MN, USA), according to the manufacturer’s recommendations.

***RNA extraction and cDNA synthesis***

Total RNA was isolated from cells by using RNeasy Mini Kit (Quiagen) according to the manufacturer’s instructions. Total RNA was quantified spectrophotometrically, and 1 µg was treated with 1U of DNAse RNAse-free (Quiagen). cDNA synthesis was performed by using the QuantiTect Reverse Transcription Kit (QIAGEN), according to the manufacturer’s instructions. For oligo sequences, see Supplementary Materials.

***RNA expression studies (qPCR)***

Total RNA was isolated by using the RNeasy Plus Mini kit (QIAGEN, Hilden, Germany) and quantified by using a Nanodrop 2000 UV visible spectrophotometer. One microgram of mRNA was reverse transcribed into cDNA by using an QuantiTect Reverse Transcription Kit (QIAGEN), according to the manufacturer’s instructions.

Real-time qPCR was performed on cDNA by using StepOnePlus™ Real-Time PCR System (Applied bioscience) and Takyon™ ROX SYBR 2X MasterMix dTTP blue (Eurogentec). For this test, predesigned primer pairs (Supp. Table S2) were purchased from Eurogentec. Fold changes in expression were calculated by the delta Ct method using RPLP0 (36B4) gene as an endogenous control for mRNA expression. All fold changes were expressed as normalized to the untreated control. Measurements were done in triplicate.

***Immunofluorescence***

Tumor tissues were fixed in PFA (4%) for half an hour immediately after dissection, then placed in a 30% sucrose solution overnight. Tumor tissues were frozen in OCT, then serially sectioned at five micrometers thick and allowed to sit at room temperature for half an hour to prepare for the immunostaining procedure. Prior to the application of the primary antibody, tissue sections were blocked for 60 minutes in phosphate-buffered saline (PBS) containing 1% bovine serum albumin (BSA), 5% horse serum, and 0.2% Triton. Incubation with primary antibodies was carried out either for one hour at room temperature or overnight at +4 °C. Negative controls were treated with blocking solution without the primary antibody. Incubation with fluorochrome-conjugated secondary antibodies (anti-rabbit AlexaFluor 488 and anti-rat AlexaFluor 647), specific to each primary antibody, was performed for 60 minutes at room temperature in the dark. DAPI was used to visualize nuclei.

***3D Microvessel***

To prepare a 3D microvessel with tumor cells in collagen gel, in-house polydimethylsiloxane (PDMS)-based chips (25 mm × 25 mm × 5 mm; width × length × height) were used, as described previously (1,2). A total of 10,000 tumor cells were mixed with a neutralized collagen solution (2.4 mg/mL) and added to the central chamber of the device. A BSA-coated acupuncture needle (200 μm in diameter; Seirin, Shizuoka, Japan) was inserted into the chip to create a lumen structure within the collagen gel. The devices were then incubated at 37°C under a humidified atmosphere of 5% CO₂ for 60 minutes to allow collagen gel polymerization. The collagen gels remained in the side chambers, and the inserted needles were removed. To form microvessels, HDLECs (Promocell) were seeded into the collagen gel channel at a density of 1 × 10⁷ cells/mL. After allowing microvessel formation for 10 minutes, the medium was replaced with EGM-2 (Lonza) for subsequent culture of the microvessel and tumor organoids within the collagen gels. Vessel permeability was assessed after 24 hours of co-culture, as previously described. Briefly, 70 kDa FITC-dextran (Sigma-Aldrich) was injected into the vessel lumen, and the rate of dextran leakage from the lumen into the surrounding culture medium was measured to determine endothelial permeability.

***Transcriptome Analysis***

*RNA isolation and amplification*

Total RNA was extracted from tissues lysed by TissueLyser II (QIAGEN) using phenol-chloroform extraction method with Trizol (Invitrogen) and Isopropanol (XILONG). The quality of the total RNA was controlled with concentration determination by using the Bioanalyzer 2100 (Agilent). All RNAs used in the present study had RNA Integrity Numbers (RINs) higher than 9.0.

*RNA-Sequencing*

RNA isolation, library preparation, and RNA-Seq were performed by BGI genomics (Hong Kong, China). Library preparation was performed from 1 µg of total RNA was used to prepare mRNA library using MGIEasy RNA Library Prep kit (MGI, China). Libraries were qualified on Bioanalyzer 2100 (Agilent) and quantified by PCR; single strand circle DNA (ssCir DNA) were formatted as the final library. The libraries of 9 samples were then amplified to make DNA nanoball (DNB) which and sequenced on DNBSEQ G400 sequencer (MGI, China) by pair-end 150, at least 30M clean reads was obtained for each library.

***Proteome Analysis***

*Migration on SDS-PAGE gel*

Each sample was deposited on a 10% acrylamide SDS-PAGE. After a short migration, gels were stained with colloidal blue overnight. After destaining, gels were scanned.

*Identification of peptides/proteins by mass spectrometry*

The bands of interest from the SDS-PAGE gel were cut and destained. Reduction disulfide bonds and alkylation of cysteine were performed, and proteins were finally digested overnight with trypsin. Resulting peptides were extracted from the gel, acidified and were injected on the Lumos with 146 min gradient OT/OT method. The spectra obtained were analyzed by Proteome Discoverer 3.1 with Sequest HT as the search algorithm, using a Human Uniprot database, including 2 missed cleavage sites by trypsin, carbamidomethylation of cysteine (+57 Da as static modification), oxidation of methionine (+16 Da as dynamic modification), acetylation of protein N-term (+42 Da as dynamic modification), Methionine loss (-131 Da as dynamic modification) and methionine loss/acetylation (+89 Da as dynamic modification).

***Subcutaneous xenografts***

This study was conducted in compliance with the National Charter on the ethics of animal experimentation. Our experiments were approved by the “Comité National Institutionnel d'Éthique pour l’Animal de Laboratoire (CIEPAL)” (reference: NCE/2023–823). Five million MDAMB231 cells (WT for one round of experiment and WT, Proton and Photon multil irradiated for another round) were injected, in medium containing 50% Corning® matrigel® matrix (VWR), subcutaneously, into the flank of 7-week-old NMRI-Foxn1nu/Foxn1nu female mice (Janvier Labs, Le Genest-Saint-Isle, France). Tumor volume was measured every other day with a caliper and calculated as follows:

𝑉=𝜋/6×𝐿×𝑙2

where *L* = tumor length; *l* = tumor width (in a 2D space, tangential to the mouse skin).

The experiment was stopped when the tumors of one experimental group reached a volume of 1000 mm^3^.

***Statistical analysis***

Results are expressed as mean ± SD and analyzed using GraphPad Prism 9 (RRID:SCR_002798) software. The unpaired Student’s *t* test was used to determine the difference between 2 independent groups. For multiple comparison analyses, one-way ANOVA test with Turkey’s correction was used. Evaluation of the gaussian distribution of the data was performed prior to the *t* test or ANOVA. Normal distribution of the data and variance similarity were verified using GraphPad Prism. A *P* value less than 0.05 was considered statistically significant. All experiments were performed with a minimum of *N* = 3 biological replicates and *n* = 3 technical replicates. For *in vivo* studies, two-tailed Mann-Whitney U test was utilized to compare two independent groups and the sample size was determined using the methods described by Berndtson *et al*.(2). Predetermined exclusion criteria included the absence of signal at the start of the experiment.
