# Supplementary material for: Comparative impact of proton versus photon irradiation on triple‐negative breast cancer: Role of VEGFC in tumour aggressiveness
Source: Clin Transl Med. 2025 May 21;15(5):e70330. doi: 10.1002/ctm2.70330 (PMC12095174; doi:10.1002/ctm2.70330)
Supplement: Supplementary file 2 — Supporting Information [file CTM2-15-e70330-s003.pdf]

| | MDAMB231 |
|---|---|
| Clone | Nucleotide/protein sequence |
| VEGFC WT | CAGTTACGGTCTGTGTCCAGTGTAGATGAACTCATGACTGTA<br>Q L R S V S S V D E L M T V |
| VEGFC Ctl1 | CAGTTACGGTCTGTGTCCAGTGTAGATGAACTCATGACTGTA<br>Q L R S V S S V D E L M T V |
| VEGFC Ctl2 | CAGTTACGGTCTGTGTCCAGTGTAGATGAACTCATGACTGTA<br>Q L R S V S S V D E L M T V |
| VEGFC KO1 | CAGTTACGGTCTGTGTCCAGA—AGATGACCTCATGGCTGTCCT<br>Q L R S V S R R * |
| VEGFC KO2 | CAGTTACGGTCTGTGTCCAGTGAA-A-GAACTCCTGGATGGAC<br>Q L R S V S S E R T P G W |

| | BT549 |
|---|---|
| Clone | Nucleotide/protein sequence |
| VEGFC WT | CAGTTACGGTCTGTGTCCAGTGTAGATGAACTCATGACTGTA<br>Q L R S V S S V D E L M T V |
| VEGFC Ctl1 | CAGTTACGGTCTGTGTCCAGTGTAGATGAACTCATGACTGTA<br>Q L R S V S S V D E L M T V |
| VEGFC Ctl2 | CAGTTACGGTCTGTGTCCAGTGTAGATGAACTCATGACTGTA<br>Q L R S V S S V D E L M T V |
| VEGFC KO1 | CAGTTACGGTCTGTGTCCAG--------------TGACTGTA<br>Q L R S V S * |
| VEGFC KO1 | CAGTTACGGTCTGTGTCCAG--------------TGACTGTA<br>Q L R S V S * |

**Table S1: Sarlak, S *et al.***
