# Supplementary material for: Comparative impact of proton versus photon irradiation on triple‐negative breast cancer: Role of VEGFC in tumour aggressiveness
Source: Clin Transl Med. 2025 May 21;15(5):e70330. doi: 10.1002/ctm2.70330 (PMC12095174; doi:10.1002/ctm2.70330)
Supplement: Supplementary file 3 — Supporting Information [file CTM2-15-e70330-s001.pdf]

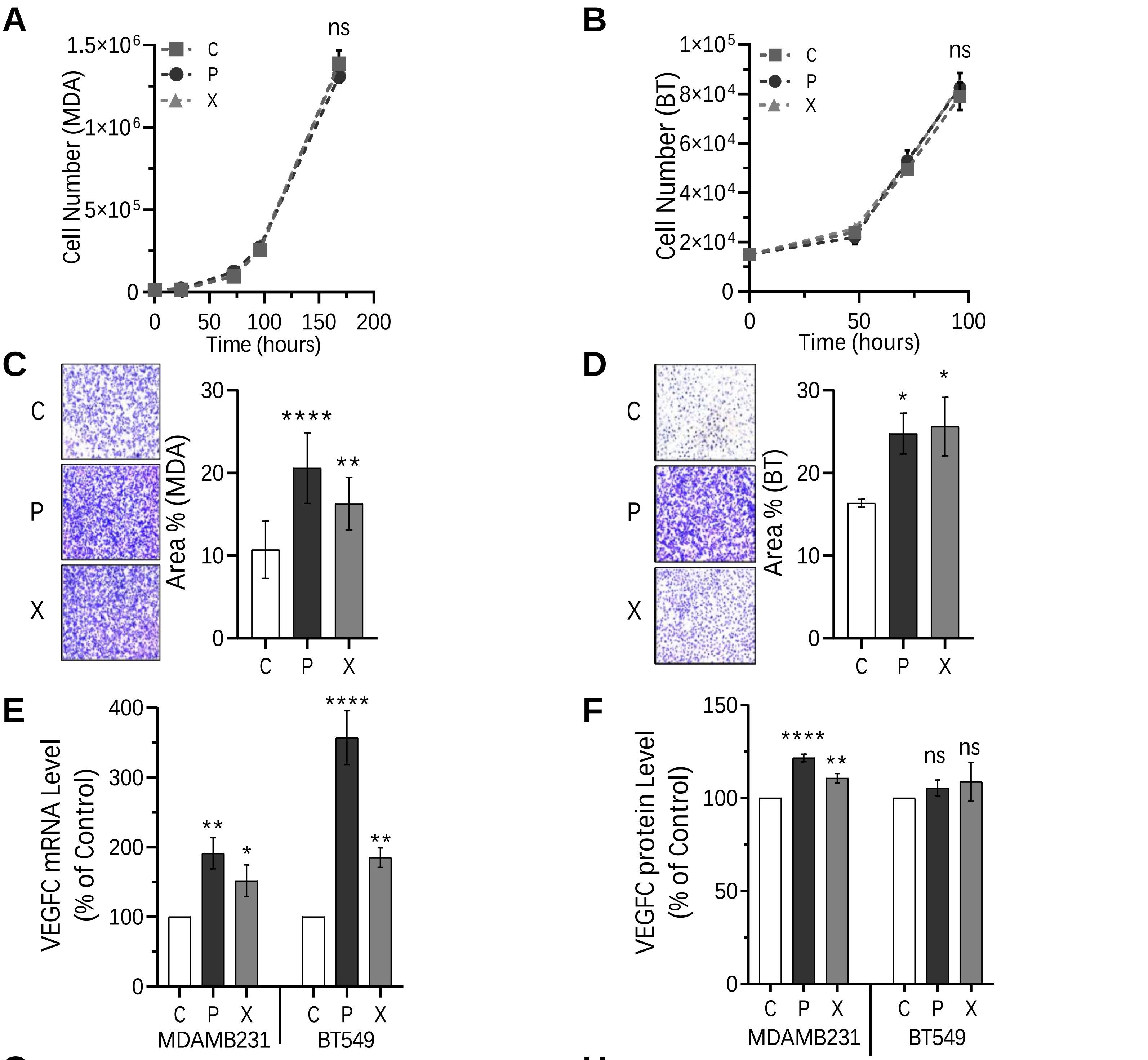

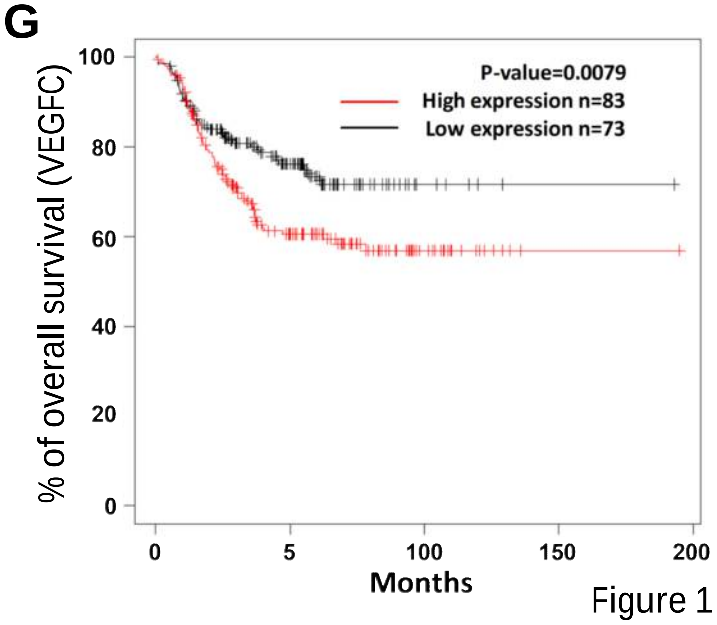

H

	MDAMB231		BT549	
	P	X	P	X
VEGFA	ns	***	ns	ns
VEGFC	****	**	**	**
Ang2	***	*	*	ns
Artemin	***	****	***	*
IGFBP2	****	*	ns	****
TGFB	**	*	*	ns
FAP	**	*	ns	*

Figure 1: Sarlak, S *et al.*

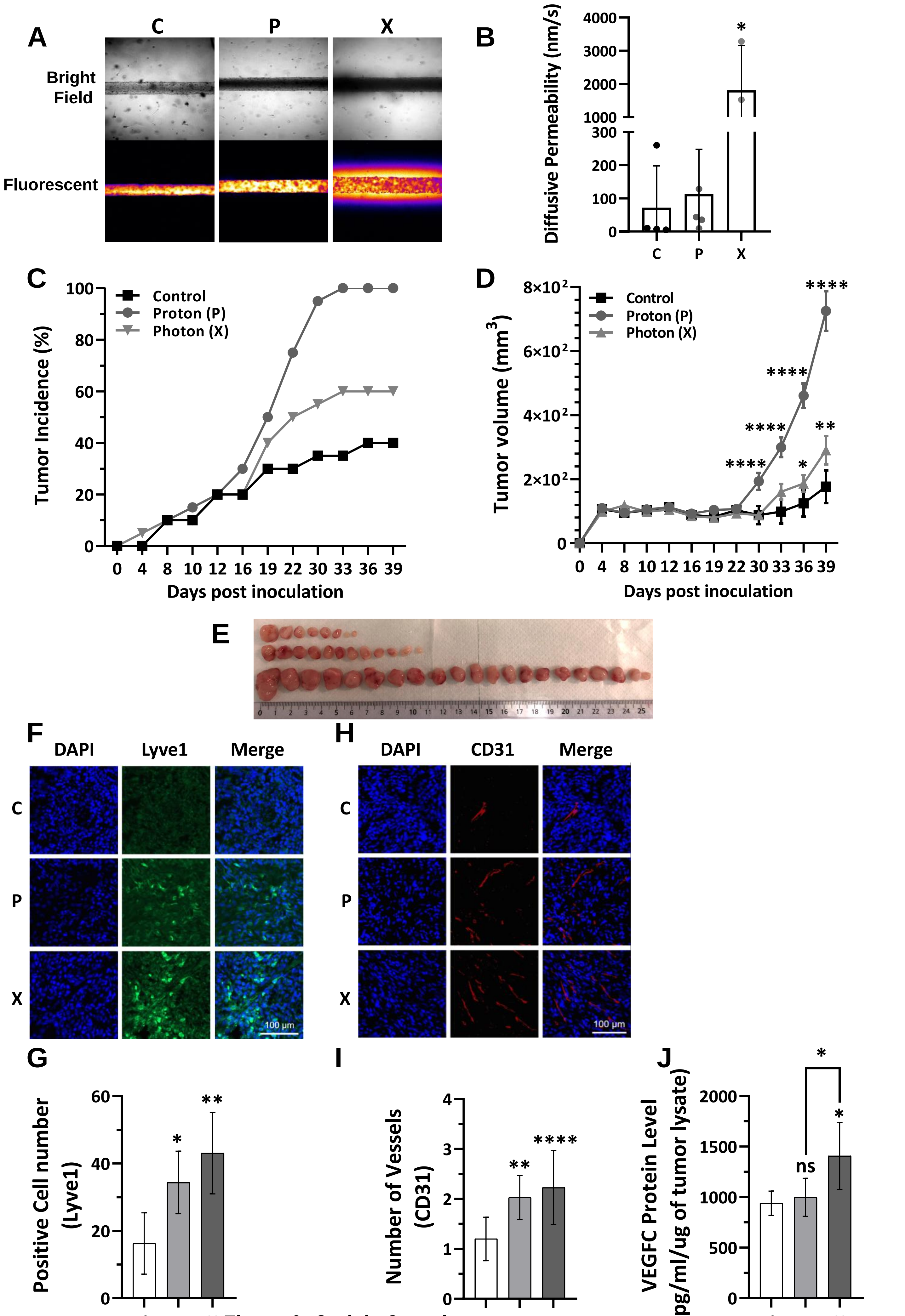

Figure 2: Sarlak, S *et al.*

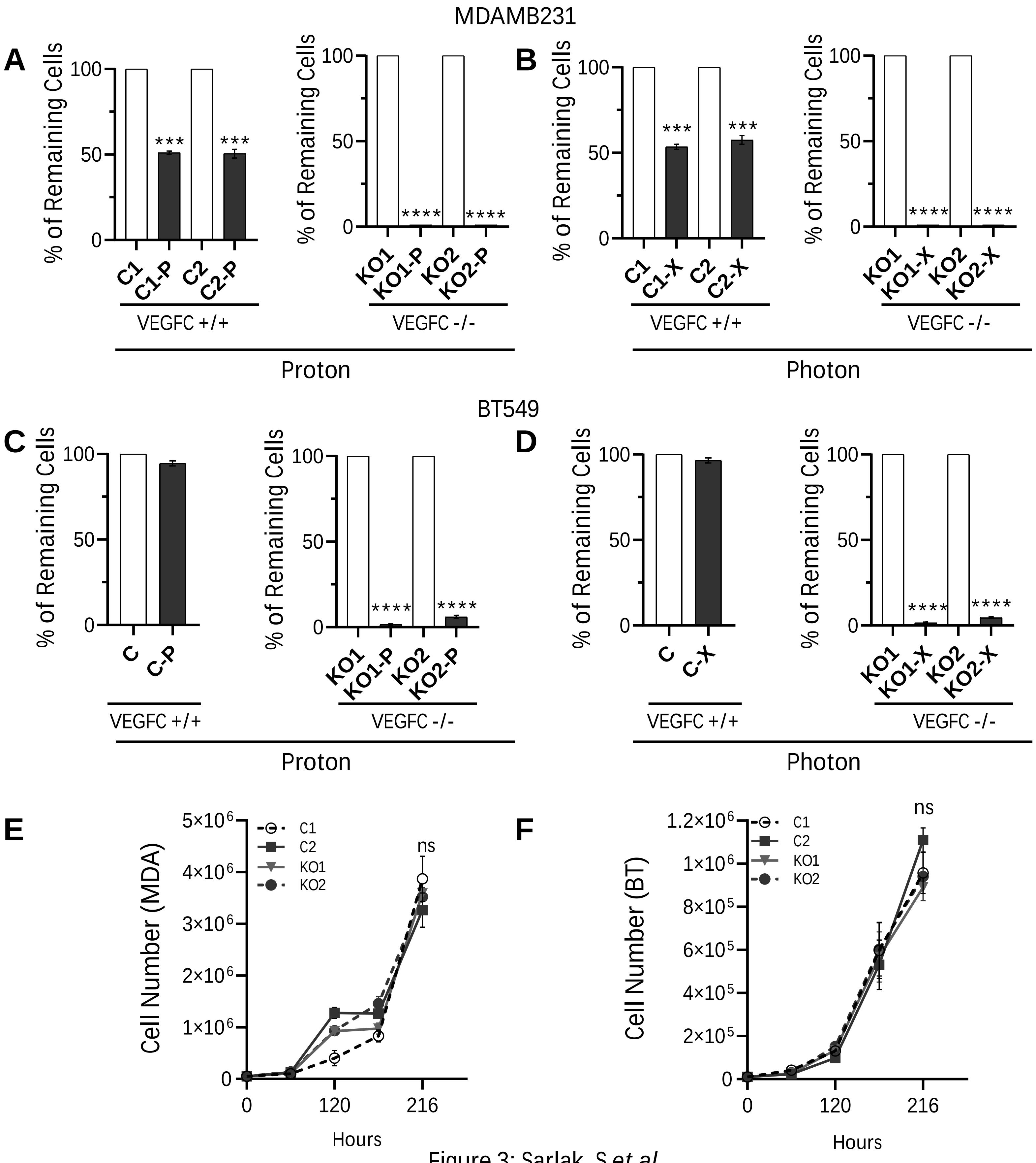

Figure 3: Sarlak, S *et al.*

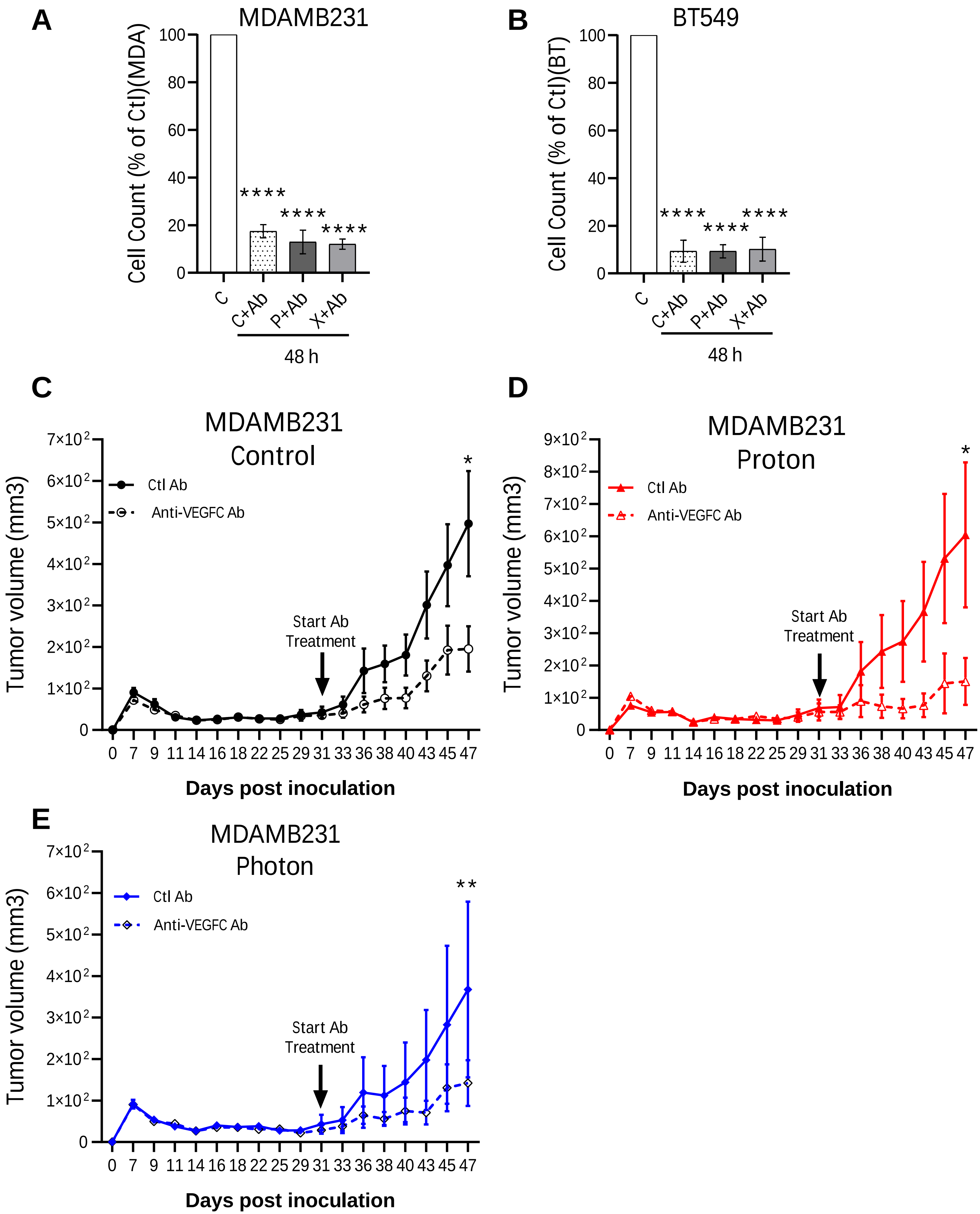

Figure 4: Sarlak, S *et al.*

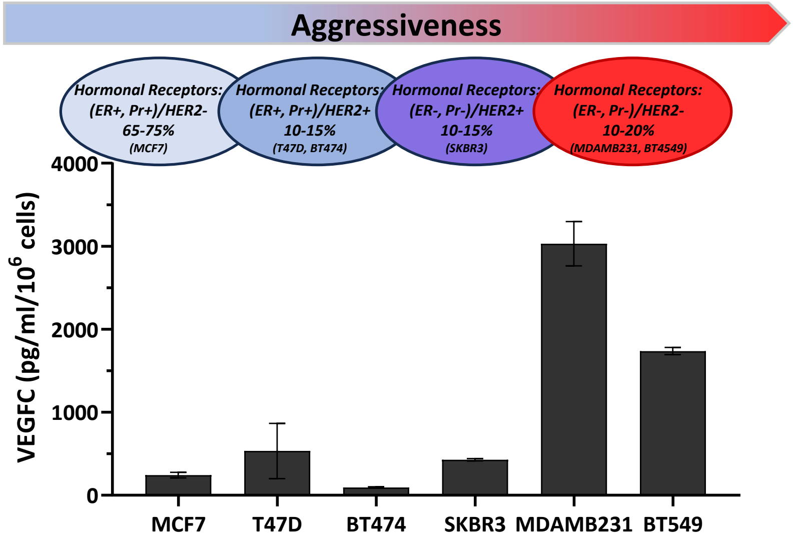

Figure S1: Sarlak, S *et al.*

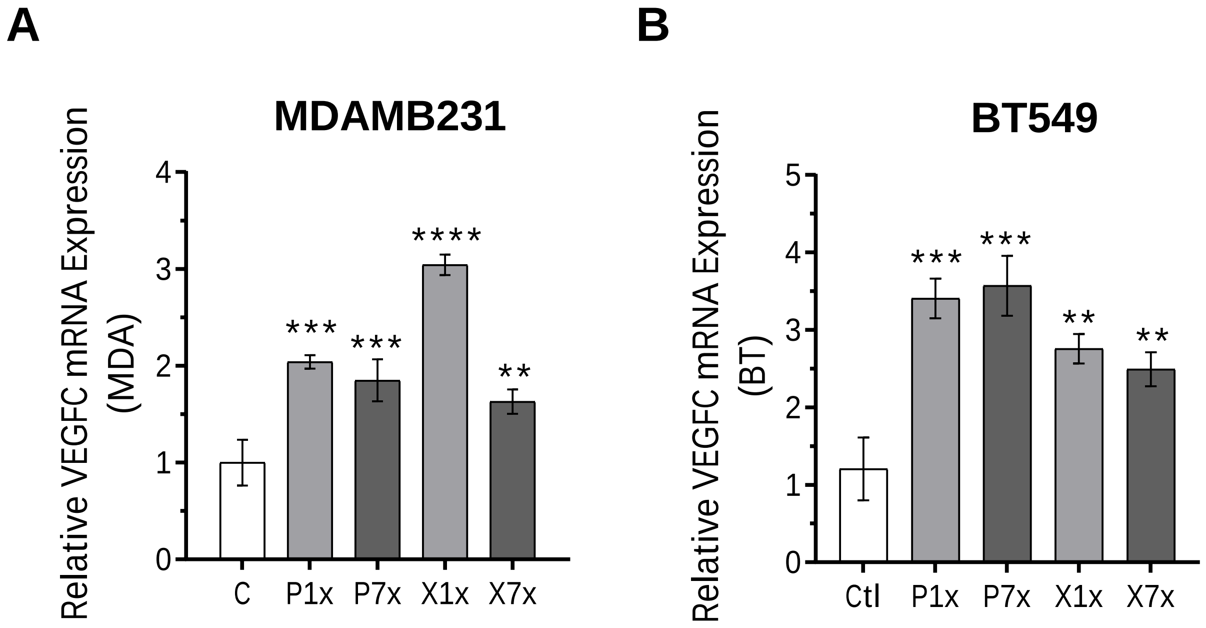

Figure S2: Sarlak, S *et al.*

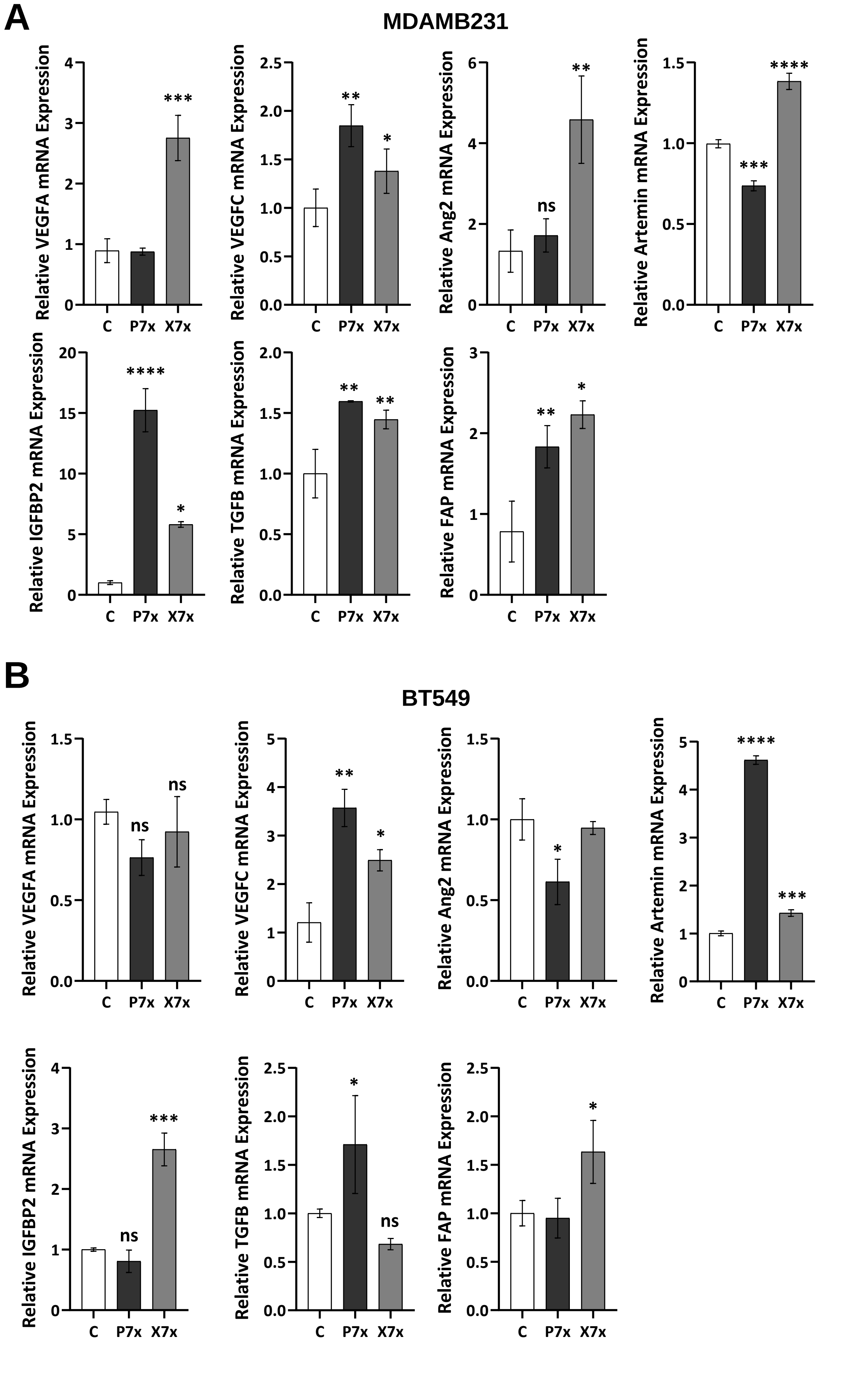

Figure S3: Sarlak, S. *et al*

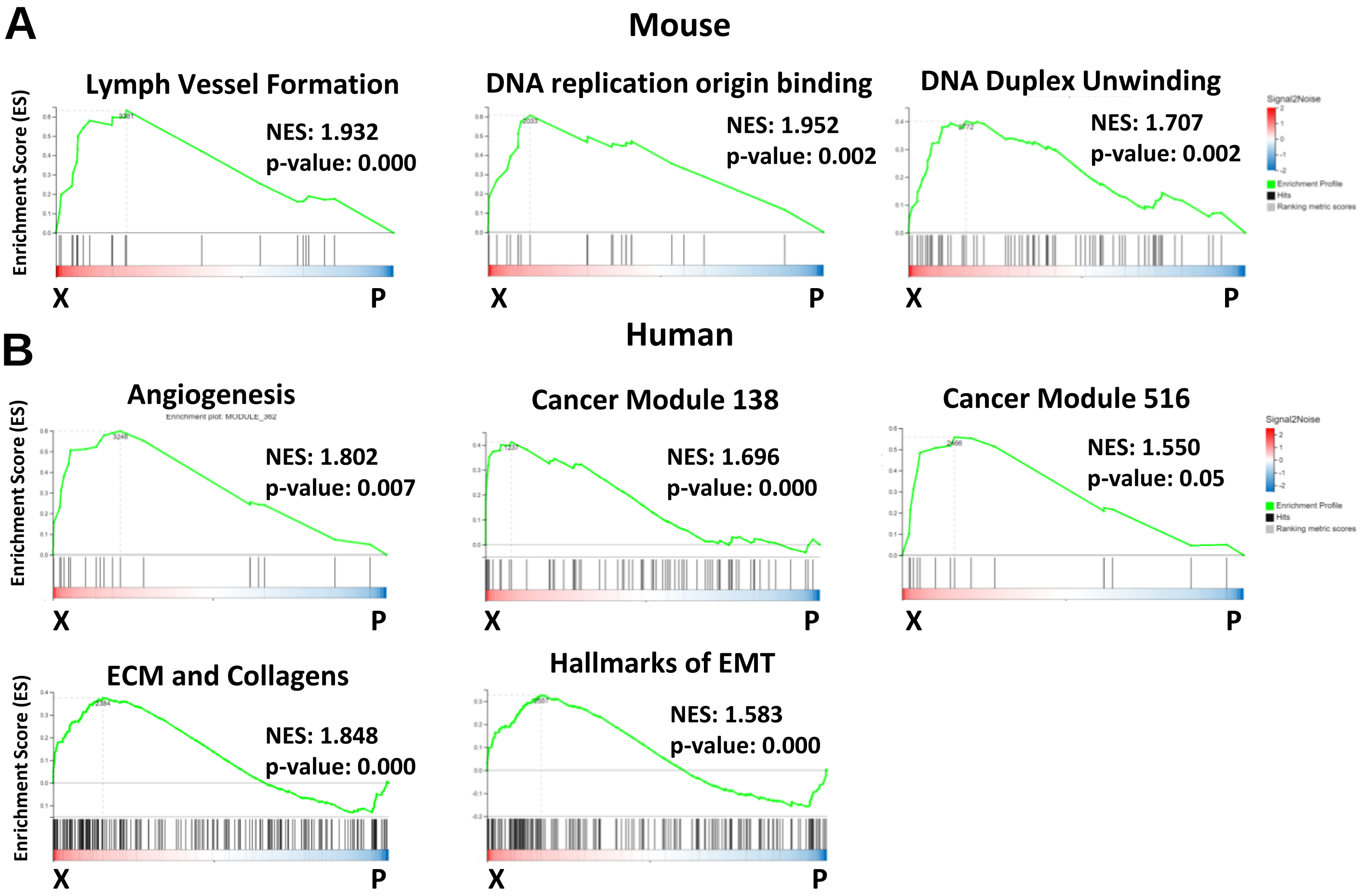

Figure S4: Sarlak, S *et al.*

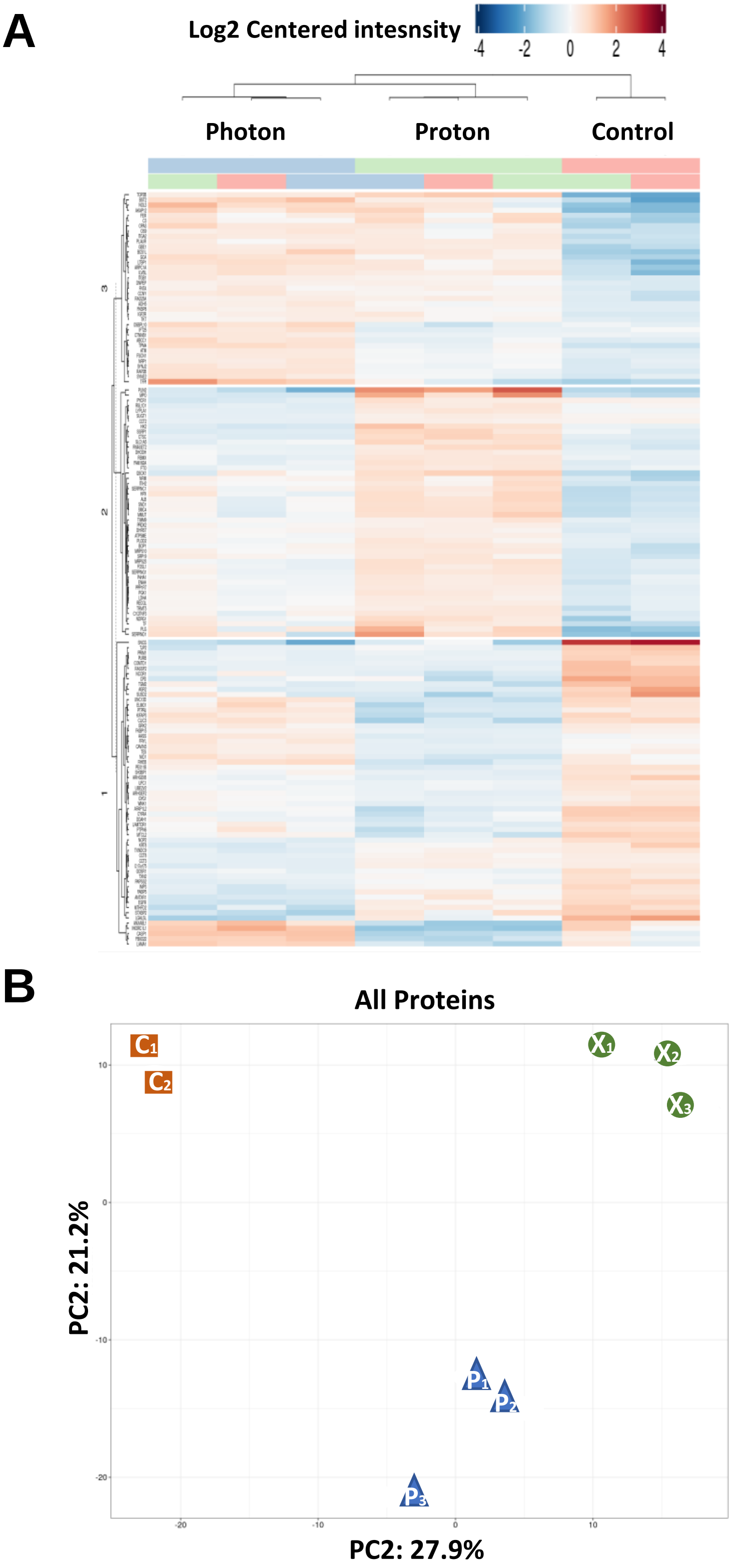

Figure S5: Sarlak, S *et al.*

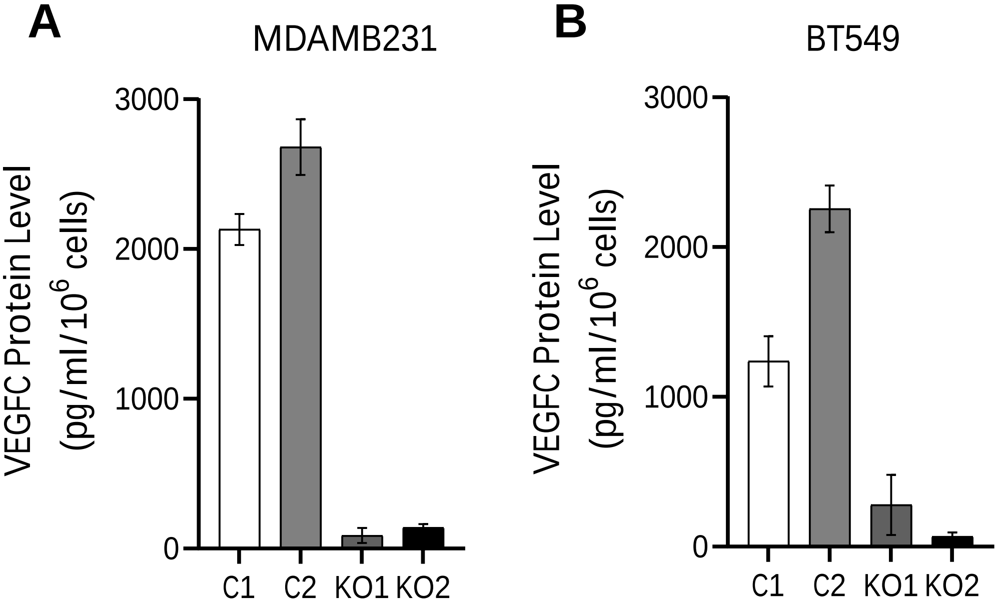

Figure S6: Sarlak, S. *et al*

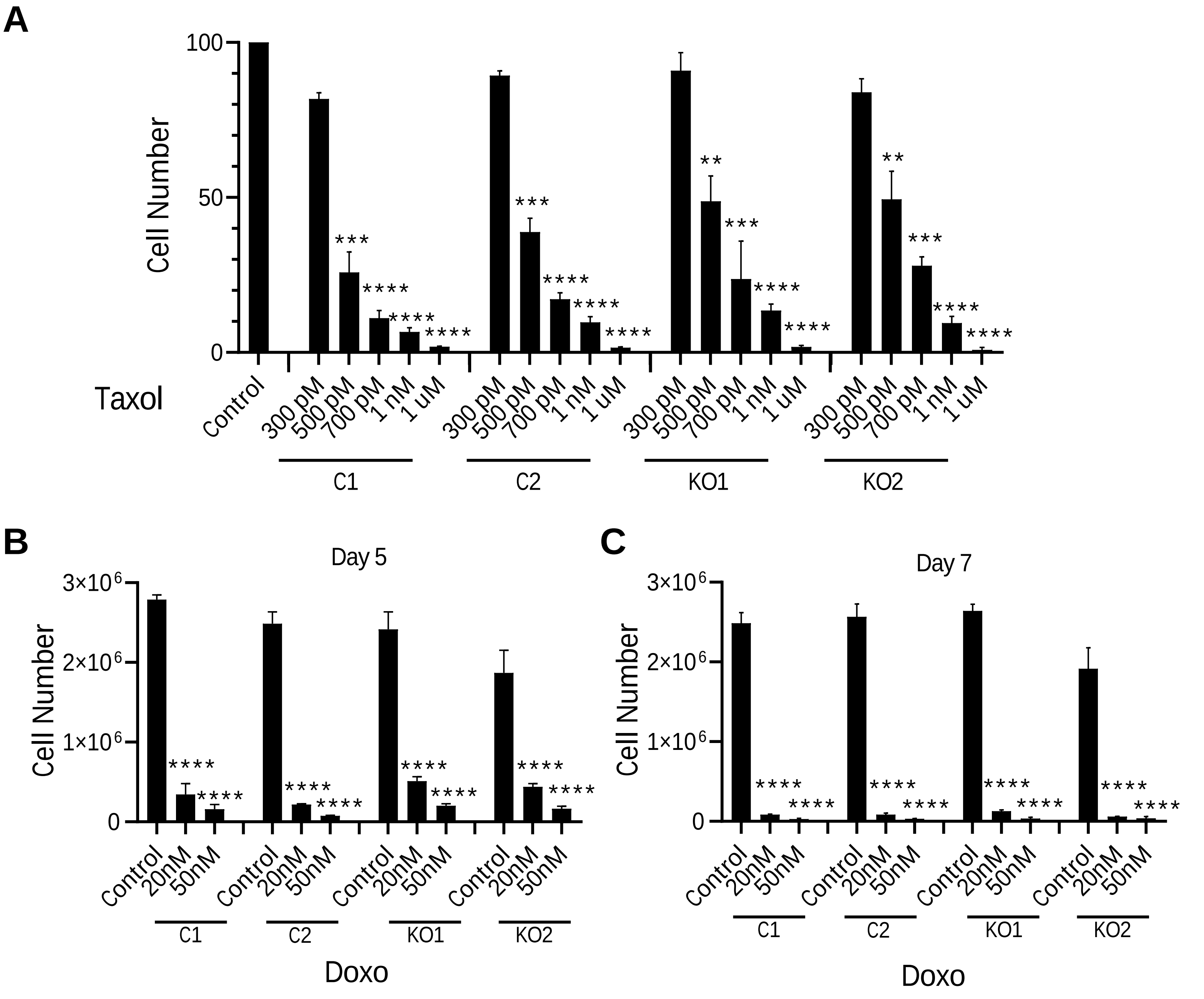

Figure S7: Sarlak, S *et al.*

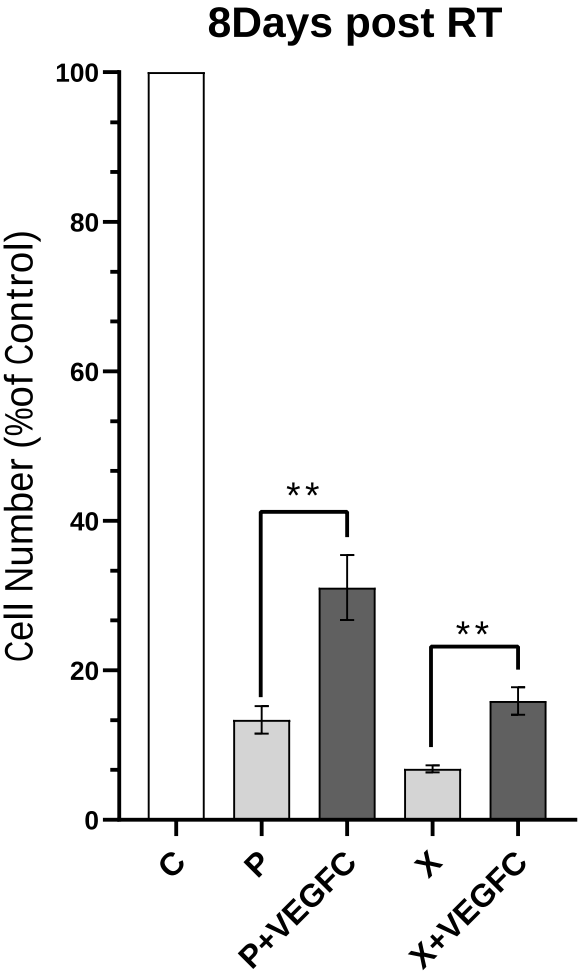

Figure S8: Sarlak, S *et al.*

	**MDAMB231**
Clone	Nucleotide/protein sequence
VEGFC WT	CAGTTACGGTCTGTGTCCAGTGTAGATGAACTCATGACTGTA<br>Q L R S V S S V D E L M T V
VEGFC Ctl1	CAGTTACGGTCTGTGTCCAGTGTAGATGAACTCATGACTGTA<br>Q L R S V S S V D E L M T V
VEGFC Ctl2	CAGTTACGGTCTGTGTCCAGTGTAGATGAACTCATGACTGTA<br>Q L R S V S S V D E L M T V
VEGFC KO1	CAGTTACGGTCTGTGTCCAGA—AGATGACCTCATGGCTGTCCT<br>Q L R S V S R R *
VEGFC KO2	CAGTTACGGTCTGTGTCCAGTGAA-A-GAACTCCTGGATGGAC<br>Q L R S V S S E R T P G W

	**BT549**
Clone	Nucleotide/protein sequence
VEGFC WT	CAGTTACGGTCTGTGTCCAGTGTAGATGAACTCATGACTGTA<br>Q L R S V S S V D E L M T V
VEGFC Ctl1	CAGTTACGGTCTGTGTCCAGTGTAGATGAACTCATGACTGTA<br>Q L R S V S S V D E L M T V
VEGFC Ctl2	CAGTTACGGTCTGTGTCCAGTGTAGATGAACTCATGACTGTA<br>Q L R S V S S V D E L M T V
VEGFC KO1	CAGTTACGGTCTGTGTCCAG--------------TGACTGTA<br>Q L R S V S *
VEGFC KO1	CAGTTACGGTCTGTGTCCAG--------------TGACTGTA<br>Q L R S V S *

Table S1: Sarlak, S *et al.*
